# Aggregates of bacteriophage 0305φ8-36 seed future growth

**DOI:** 10.1186/1743-422X-4-131

**Published:** 2007-12-04

**Authors:** Philip Serwer, Shirley J Hayes, Karen Lieman

**Affiliations:** 1 Department of Biochemistry, The University of Texas Health Science Center, San Antonio, Texas 78229-3900, USA

## Abstract

Lytic bacteriophage 0305φ8-36 forms visually observed aggregates during plaque formation. Aggregates intrinsically lower propagation potential. In the present study, the following observations indicate that lost propagation potential is regained with time: (1) Aggregates sometimes concentrate at the edge of clear plaques. (2) A semi-clear ring sometimes forms beyond the plaques. (3) Formation of a ring is completely correlated with the presence of aggregates at the same angular displacement along the plaque edge. To explain this aggregate-derived lowering/raising of propagation potential, the following hypothesis is presented: Aggregation/dissociation of bacteriophage of 0305φ8-36 is a selected phenomenon that evolved to maintain high host finding rate in a trade-off with maintaining high rate of bacteriophage progeny production. This hypothesis explains ringed plaque morphology observed for other bacteriophages and predicts that aggregates will undergo time-dependent change in structure as propagation potential increases. In support, fluorescence microscopy reveals time-dependent change in the distance between resolution-limited particles in aggregates.

## Findings

The life cycle of a virus incorporates evolutionary compromises (trade-offs) between propagation of the virus and propagation of its host [1-4]. These trade-offs are thought to be a component in bacteriophage/host co-evolution, thought to be a major factor in bacterial speciation [5,6]. Intracellular bacteriophage sequestering via lysogeny [6-8] is one apparent source of trade-off in favor of host finding. For example, bacteriophage λ lysogeny is promoted by raising the bacteriophage concentration [9,10]. This observation supports the idea that bacteriophage λ evolved lysogeny, in part, to avoid severe host depletion, i.e., to increase the long-term host finding rate. Lytic bacteriophages do not have access to trade-off of this type.

However, in the case of lytic bacteriophages, extracellular sequestering by a heterogeneous environment can be a significant source of trade-off to increase host-finding rate [11]. In theory, sequestering by lytic bacteriophage aggregation would be yet another source of such trade-off, potentially specific only for bacteriophage concentrations high enough to threaten host extinction. However, extensive aggregation has not been observed for well studied lytic bacteriophages, until recently.

Within the past year, report was made of propagation in dilute (0.08 – 0.15%) agarose gels to isolate a lytic *Bacillus thuringiensis *bacteriophage, 0305φ8-36 [12], which forms extensive, sometimes millimeter-sized aggregates during plaque formation [13]. If aggregate formation, along with reduced virus reproduction, is a selected phenomenon, then aggregate dissociation must eventually occur and seed a phase of increased bacteriophage reproduction. However, no current observation reports aggregate-derived seeding of 0305φ8-36 propagation in either environmental or laboratory culture. The present study investigates the possible existence of such seeding within a 0305φ8-36 plaque.

To form cm-sized plaques that do not overlap, bacteriophage 0305φ8-36 was inoculated at four locations in a pre-formed, upper layer, 0.1% agarose gel that contained both host cells and growth medium; gelation had been performed at room temperature (25 ± 3°C). This gel was above a lower layer 1.0% agar gel in a Petri dish [12]. After incubation at room temperature, four clear, 1.2 – 1.6 cm plaques were visible by about 8 hr (not shown). When the plaques were incubated for another 24 hours, they became larger, 3.8 – 4.8 cm, and acquired the following additional features.

**Figure 1:**
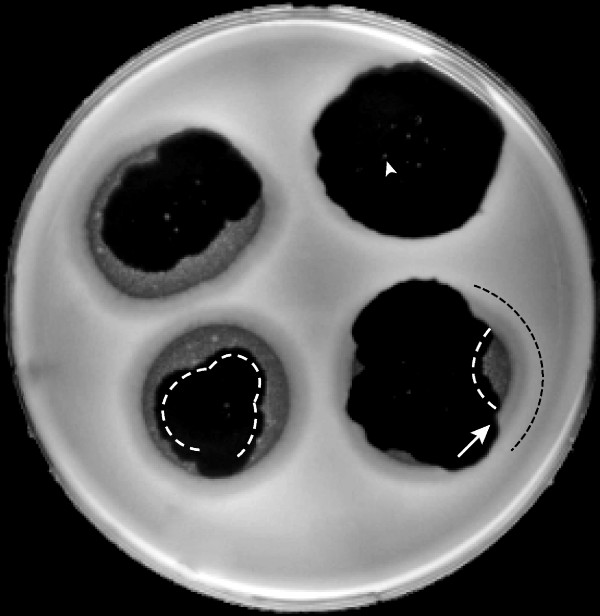
In-plaque aggregation/dissociation of bacteriophage 0305φ8-36. A 0.1% agarose overlay was mixed with host cells, poured over a 1.5% agar gel and gelled in a Petri plate, as described in the text. Four plaques were initiated by stabbing and the Petri plate was incubated for 32 hr. at room temperature (25 ± 3°C). Light scattering was photographed. The white dashed lines indicate opaque zone segments that are near the edge of a mostly clear plaque. The black dashed line indicates a semi-clear ring segment. The arrowhead indicates an opaque spot. The arrow indicates a comparatively turbid region between clear plaque and semi-clear ring.

Most plaques had internal, roughly circular opaque spots that superficially resembled bacteriophage-resistant host colonies, but are known to be aggregates of bacteriophage particles [13]. The distribution of the opaque spots varied. For example, the plaque in the upper right quadrant in Figure 1a has a few circular, ~1 mm in diameter, opaque spots scattered around the center; one is indicated with an arrowhead. The plaque in the lower left quadrant has these central opaque spots, but also (unlike the upper right plaque) has much more numerous, close-packed, sometimes merged, 1–2 mm spots that, in total, occupy a much larger "opaque zone" near the outer edge of the plaque; the inner edge of the opaque zone is indicated with a white dashed line. One small segment is missing from this zone, as indicated by interruption of the dashed line. The reason for variation in the distribution of opaque zones is not known.

Of the four plaques in Figure 1, all but the upper right plaque have opaque zones near the plaque edge. All three plaques with opaque zones, but not the upper right plaque, also have a semi-clear ring that is (a) interrupted in some places and (b) parallel to and outside of the edge of the clear plaque. A black dashed line at the right of the lower right plaque indicates a segment of this semi-clear ring. The key observation is the following: Wherever a semi-clear ring exists, an opaque zone also exists at the same angular displacement along the circumference of the plaque (ring/zone correlation). This observation is made by inspecting the plaques of Figure 1 and was also made for all of over 100 other plaques (not shown). A ring extends a little further than the corresponding opaque zone, along the plaque edge. In Figure 1, the ring/zone correlation is most dramatic for the lower right plaque, which has three interruptions in the semi-clear ring and the same three interruptions in the opaque zone. The ring/zone correlation implies cause-effect between ring and zone. Furthermore, the turbid zones must be the cause of the semi-clear rings because the turbid zones are in a part of the plaque that formed earlier than the semi-clear rings.

If the bacteriophage that lyses cells in the semi-clear ring is the original bacteriophage, then the bacteriophage particles in a turbid zone have seeded propagation in the conjugate semi-clear ring. By the following criteria, the bacteriophages in the semi-clear ring are, indeed, particles of the original bacteriophage, not particles of a mutant or an induced prophage or a contaminant bacteriophage:

(a) After re-propagation of 0305φ8-36 from opaque spot, opaque zone, clear interior, semi-clear ring and intermediate regions, plaques were the same as plaques after the original propagation within the limits of variability found for the original propagation and illustrated in Figure 1 (data not shown). (b) By pulsed field gel electrophoresis, DNA from the various regions of plaques migrates at the rate of mature 218.948 Kb 0305φ8-36 DNA [14]. (c) Finally, analysis of the complete nucleotide sequence of 0305φ8-36's genome reveals no lysogeny module [15].

Thus, the aggregates seed growth of 0305φ8-36 in the semi-clear ring. How, then, does this seeding effect occur when an opaque zone is separated from its conjugate semi-clear ring by 1–3 mm of plaque-supporting clear gel (Figure 1) that also has aggregates and the same total concentration of bacteriophage particles, within a factor of 2 [13,14]? To provide a likely answer to this question, we note that (a) formation of an aggregate, by necessity, initially causes propagation potential to drop and (b) aggregates in opaque zones are older than aggregates in the clear zone further away from the center of the plaque. Therefore, we hypothesize that, as bacteriophage 0305φ8-36 aggregates age, something about them changes to make constituent bacteriophages more prone to dissociate and infect cells. That is the likely answer to the above question.

In fact, large (> 1 μm) 0305φ8-36 aggregates are already known to increase in elasticity with time [13]. To further investigate possible time-dependent changes of these aggregates, we investigated the time-dependence of the separation of resolution-limited, aggregate-associated particles after dissection of aggregates from a single plaque at a single radius, but at different times. This study revealed that the distance between the particles decreased from 1.6 ± 0.97 μm at 10.5 hr. to 0.54 ± 0.4 μm at 32 hr. and 0.2 ± 0.2 μm at 50 hr (images not shown). Details are not known for how this decrease in distance causes infective particle dissociation. Perhaps, (a) the decrease in inter-particle distance places stress on the tail and associated fibers that bridge particles in an aggregate and (b) this stress is relieved by dissociation.

In analogy with 0305φ8-36 plaques, ringed plaques (sometimes called either "target" or "bulls-eye" plaques) have previously been observed, although no explanation for this morphology was presented [16,17]. The present study raises the possibility that plaque rings, in general, are caused by aggregation/dissociation as hypothesized here. One of the previous ringed plaque-forming bacteriophages has, in fact, been shown to aggregate by electron microscopy [17].

Current theories of host evolution assume no change in bacteriophage reproduction capacity as the concentration of a lytic bacteriophage increases (see, for example, refs [18,19]). We propose that concentration-dependent, aggregation-based 0305φ8-36 sequestering, followed by dissociation, is (a) part of an evolutionary trade-off for the purpose of increasing host finding rate and (b) a phenomenon that changes the concept of how some lytic bacteriophages both optimize their reproduction [19] and participate in host evolution.

## Competing interests

The author(s) declare that they have no competing interests.

## Authors' contributions

PS interpreted the data and wrote the manuscript. SJ Hayes and KL performed the experiments.
